# Intelligibility Evaluation of Pathological Speech through Multigranularity Feature Extraction and Optimization 

**DOI:** 10.1155/2017/2431573

**Published:** 2017-01-17

**Authors:** Chunying Fang, Haifeng Li, Lin Ma, Mancai Zhang

**Affiliations:** ^1^School of Computer Science and Technology, Harbin Institute of Technology, Harbin, China; ^2^School of Computer and Information Engineering, Heilongjiang University of Science and Technology, Harbin, China

## Abstract

Pathological speech usually refers to speech distortion resulting from illness or other biological insults. The assessment of pathological speech plays an important role in assisting the experts, while automatic evaluation of speech intelligibility is difficult because it is usually nonstationary and mutational. In this paper, we carry out an independent innovation of feature extraction and reduction, and we describe a multigranularity combined feature scheme which is optimized by the hierarchical visual method. A novel method of generating feature set based on *S*-transform and chaotic analysis is proposed. There are BAFS (430, basic acoustics feature), local spectral characteristics MSCC (84, Mel *S*-transform cepstrum coefficients), and chaotic features (12). Finally, radar chart and *F*-score are proposed to optimize the features by the hierarchical visual fusion. The feature set could be optimized from 526 to 96 dimensions based on NKI-CCRT corpus and 104 dimensions based on SVD corpus. The experimental results denote that new features by support vector machine (SVM) have the best performance, with a recognition rate of 84.4% on NKI-CCRT corpus and 78.7% on SVD corpus. The proposed method is thus approved to be effective and reliable for pathological speech intelligibility evaluation.

## 1. Introduction

Pathological speech usually refers to speech distortion resulting from illness or other physical biological insults to the production system. It is difficult to evaluate pathological speech intelligibility. Over the years, there has been considerable interest in offering objective and automated schemes to measure and classify pathological speech quality, hoping that both improved accuracy and reliability in the processing can be offered. Researchers have extensively studied the different features of the pathological speech evaluation. Kim et al. performed feature-level fusions and subsystem decision fusions for the best classification performance (73.5% for unweighted) on NKI-CCRT corpus [1]. Shama analyzed the sustained vowels and extracted the HNR and the critical-band energy spectrum to different pathological and healthy voice [2]. Gelzinis et al. researched on diseases of the larynx and extracted the fundamental frequency, perturbation coefficient, and linear prediction coefficient of pathological speech features [3]. Zhou et al. extracted time-frequency domain modulated characteristics to analyze pathological voice; a recognition rate of 68.3% is achieved based on NKI-CCRT corpus [4]. Arjmandi et al. extracted some widely used long-time acoustic parameters, such as shim, jitter, and HNR, to develop an automatic pathological voice computerized system [5]. Previous studies indicate that the voice change detection can be carried out by long-term acoustic parameters; each individual voice utterance can be quantified by a single vector. These long-time parameters are generally calculated by averaging local time perturbations. In our study, we describe an automatic intelligibility assessment system which extracts information visualization features by capturing the relation of feature of pathological speech. It may require high-dimensional acoustic features in order to capture the wide variability of sources and patterns in pathological speech. Thus, the difference granularity level pathological features are extracted; firstly, the common basic acoustic features are extracted from vocal organ lesion; it is widely recognized that the acoustic signal itself contains information about the vocal tract and the excitation waveform. Secondly, Mel frequency cepstral coefficients can be estimated by using a nonparametric fast Fourier transform, which are more dependent on high-pitched speech resulting from loud or angry speaking styles [6]. Stock proposed *S*-transform in 1996, which can be regarded as the combination of wavelet transform and short time Fourier transform [7]. Thus, we proposed MSCC (Mel *S*-transform cepstrum coefficients) features to solve the problem of time-varying dynamic pathological speech. However, pathologies speech is a fairly complex task; some of these parameters are based on an accurate estimation of the fundamental frequency. More modern approaches have been devised; linear model is not suitable to explain nonlinear characteristics. Thus, thirdly, some of the authors have also proposed nonlinear signal processing methods of the same task [8, 9]. Airflow propagation through the human's vocal tract is more likely to follow the fluid dynamic rules which lead to nonlinear models [10]; furthermore, chaos theory has been used as a powerful tool to analyze nonlinear systems [11, 12]. Therefore, the three nonlinear chaotic features can be extracted, which are the largest Lyapunov exponent, approximate entropy, and Lempel-Ziv complexity [13]. Finally, we proposed a novel hierarchical visual feature fusion method which is based on *F*-score and radar chart to optimize features set and improve system performance.

Section 2 describes a joint feature extraction process; a novel MSCC feature is computed based on *S*-transform and other common features are extracted. In Section 3, a new optimization method of joint feature set is proposed as a new method based on *F*-score and radar chart. In Section 4, the lower-dimensional feature space will be eventually performed, and speech examples from NKI-CCRT and SVD corpus are considered [14]. MSCC is similar to MFCC. We compare MSCC with MFCC, by means of *F*-score, to distinguish the ability to reduce features between normal and pathological voices in the experiments and compare the other joint feature set. Finally, conclusions are drawn and future directions are indicated in Section 5.

## 2. Multigranularity Pathological Speech Feature Extraction

### 2.1. Basis Acoustic Feature

We observed that vocal organ lesion speakers often have difficulty in pronouncing a few specific sounds, which result in abnormal prosodic and intonational shape. In order to reflect different aspects of pathological speech, we applied the following features to capture the differences between normal and pathological speech as shown in Table 1.

Voice quality features, such as fundamental frequency perturbation, shimmer, and harmonic noise ratio, are popularly used in vocal disorder assessment. Moreover, the relevant characteristics of the spectrum shape change channels (vocal tract) and vocal movement (articulator movements) can accurately reflect the substantial voice disorders changes, such as various polyps, cancer, and other sound systems [15]. There are a large number of studies mainly focused on the accurate measurement of the fundamental parameters of the previous researches, such as fundamental frequency, jitter, shimmer, amplitude perturbation quotient, pitch perturbation quotient, harmonics-to-noise ratio, and normalized noise energy. In this article, the long-time and short-time 430-dimensional acoustic parameters (basic acoustics feature set, BAFS) are extracted according to the previous studies in Table 1 [5].

### 2.2. Local Spectrum Feature Based on *S*-Transform (MSCC)

Pathological speech signal is nonstationary and mutational in time-frequency domain; in this paper, MSCC is proposed based on *S*-transform.

Let *x*(*t*) denote continuous speech signal, where *t* = *n*Δ_**T**_, Δ_**T**_ is the sampling interval, and *x*(*t*) sample sequence *x*[*n*] can be expressed as *x*[*n*] = *x*(*n*Δ_**T**_), *n* = 0,1, 2,…, *N* − 1. The *x*[*n*]  *S*-transform can learn from discrete Fourier transform calculation. The *x*[*n*] Fourier transform is

(1) $$\begin{array}{c} X\left[\;k\;\right]=\frac{1}{N}\overset{N-1}{\underset{k=0}{\sum }}x\left[\;n\;\right]e^{-2\pi jkn/N}, \end{array}$$

where *k* = 0,1 …, *N* − 1.

The discrete *S*-transform *x*[*n*] is computed by FFT:

(2) Sh,k=∑m=0N−1Xm+keh2πmj/N−2π2m2/n2,k≠0,Sh,0=1N∑m=0N−1Xm,k=0,

where *h*, *m* = 0,1, 2,…, *N* − 1.

The sampling sequence *x*[*n*] of continuous signal *x*(*t*) is converted into the *N∗N* complex time-frequency matrix by *S*-transform from (2), in which the row corresponds to time and the column corresponds to frequency.

**Figure 1:**
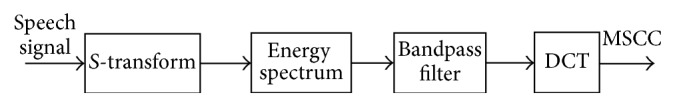
MSCC extraction based on *S*-transform.

MSCC is proposed based on *S*-transform, shown in Figure 1; the *S*-transform method reflects the human auditory Mel spectrum characters.

MSCC extraction process is as follows; *x*[*n*] is the input, and the output is *C*_1_, *C*_2_,…, *C*_*L*_; FrameLen represents the length of the frame.

(1) Framing: framing *x*[*n*] in FrameLen.
(2) *S*-Transform: transform matrix **S** is got by *S*-transform (2).
(3) Energy spectrum: energy spectra are obtained based on step (2).
(4) Bandpass filter: the 26 filter banks are constructed.
Log energy is calculated for each time in each filter bank:
  (3) $$\begin{array}{c} x^{\prime }\left(\;h,m\;\right)=\ln\left(\;\overset{N-1}{\underset{k=0}{\sum }}{\left|\;S\left[\;h,k\;\right]\;\right|}^{2}H_{m}\left(\;k\;\right)\;\right),\;0\leq m<M, \end{array}$$
where *S*[*h*, *k*] is spectrum by *S*-transform in *h*Δ_**T**_, *x*′(*h*, *m*) is the *m* filter output in *h*Δ_**T**_, and *H*_*m*_(*k*) is the frequency response of triangle filters.
(5) Discrete cosine transform (DCT): discrete time mapping cepstrum domain in the *L* MSCC coefficients is got:
  (4) Ch,n=∑m=1Mx′h,mcos⁡πnm−0.5M,1≤n≤L.

### 2.3. Chaotic Features (CF)

The chaotic-based features are presented in the previous sections, and anomalies in pathological voices stem from malfunctions of some parts of the voice production system. Speech signal has fractal characteristics; chaotic phenomena can occur during speech production when the vocal organ is within a lesion. Traditional acoustic parameters are very effective to analyze cycle speech signal, which have certain limitations on analyzing noncycle and chaotic signals. Chaotic features provide useful information on distinguishing normal and pathological voices. Therefore, three nonlinear chaotic features (CF) can be extracted, which are the largest Lyapunov exponent to measure the speech signal chaotic degree, approximate entropy to measure speech signal complexity, and Lempel-Ziv complexity which is another complexity index [16, 17], where frame length is 50 ms and frame shift is 30 ms.

In this article, the largest Lyapunov exponent extraction process as an example is introduced. In order to guarantee the largest Lyapunov exponent reliability, the classic small data set algorithm is used; we get 4 statistics (mean, variance, skewness, and kurtosis), and the 526-dimensional feature set was composed of 4 statistics and the other 522 features.

Pathological speech signal *x*(*t*) = {*x*_1_, *x*_2_, *x*_3_,…, *x*_*N*_} is a one-dimensional time series, where *N* is the total number of time series; phase space is reconstructed as follows:

(5) $$\begin{array}{c} \left[\begin{array}{c} X_{1}\\ X_{2}\\ \vdots \\ X_{M} \end{array}\right]=\left[\begin{array}{cccc} x_{1} & x_{1+\tau } & \cdots & x_{1+\left(m-1\right)\tau }\\ x_{2} & x_{2+\tau } & \cdots & x_{2+\left(m-1\right)\tau }\\ \vdots & \vdots & \vdots & \vdots \\ x_{M} & x_{M+\tau } & \cdots & x_{M+\left(m-1\right)\tau } \end{array}\right], \end{array}$$

where *m* is embedding dimension, *τ* is delay, *M* is the total number of phase points, and *M* = *N* − (*m* − 1)*∗τ*.

The specific calculation steps of the small data set method are as follows:

(1) Calculated time series averaging period *p*: the spectrum is obtained by the Fourier transform. The corresponding frequency is got in the maximum amplitude. This averaging period is the reciprocal of the frequency.
(2) In the phase space *X*(*i*), the nearest neighbor $X(\hat{j})$ of *X*(*j*) is found in the case of restrictions brief separation:
  (6) $$\begin{array}{c} d_{j}\left(\;0\;\right)=\underset{j}{\min}\left\|\;X\left(\;j\;\right)-X\left(\;\hat{j}\;\right)\;\right\|,\;\left|j-\hat{j}\right|>p, \end{array}$$
where ‖·‖ represents two-norm value and *p* is the average period of time series.
(3) For each reference point, *d*_*j*_(*i*) is the distance between *X*(*j*) and $X(\hat{j})$ in the *i* discrete time:
  (7) dji=Xj+i−Xj^+i,i=1,2,…,min⁡M−j,M−j^.
(4) The Lyapunov exponents represent the initial closed orbit exponential divergence of phase space; it is assumed that the exponential divergence *λ*_1_ is got by the reference point *X*_*j*_ and the nearest neighbor $X(\hat{j})$; then,
  (8) $$\begin{array}{c} d_{j}\left(\;i\;\right)=d_{j}\left(\;0\;\right)e^{\lambda_{1}\left(i\cdot \Delta t\right)}. \end{array}$$
Both sides of the equation were taken as the logarithm:
  (9) $$\begin{array}{c} \ln d_{j}\left(\;i\;\right)=\ln C_{j}+\lambda_{1}\left(\;i\cdot \Delta t\;\right). \end{array}$$
As can be seen above, *i* ~ ln⁡*d*_*j*_(*i*) meet the linear relation of the slope *λ*_1_Δ*t*; thus,
  (10) $$\begin{array}{c} y\left(\;i\;\right)=\frac{1}{q\Delta t}\overset{q}{\underset{j=1}{\sum }}\ln d_{j}\left(\;i\;\right), \end{array}$$
where *q* is the number of nonzero *d*_*j*_(*i*) and Δ*t* is sample sampling period.
(5) Linear regression is done using the least square, and the largest Lyapunov exponent *λ*_1_ is the slope of this line:
  (11) $$\begin{array}{c} \lambda_{1}=\frac{\sum_{i}i\cdot y\left(i\right)-\overset{-}{y}\sum_{i}i}{\sum_{i}i^{2}-\overset{-}{i}\sum_{i}i}. \end{array}$$

The 526-dimensional feature set is constructed by the above three features' extraction, which are BAFS (430), MSCC (84), and CF (12).

## 3. Feature Optimization

A set of high-dimensional data is obtained after pathological speech signal feature extraction. Visual techniques and multi-information fusion idea are a high-dimensional data reduction approach; at the same time, they depict the internal structural relationship of features, which is beneficial to data classification. Radar chart has good interaction, which is able to reflect the trend of changes in a feature set and every dimensional situation. In order to express the structural characteristics among attributes, radar chart information visualization graphical feature is extracted. According to radar chart uniqueness theorem, radar chart must be unique if the input feature is restricted to a specified alignment. Therefore, the extraction of graph feature is closely related to the feature order; we introduce *F*-score method to sort the features.

### 3.1. *F*-Score Measure for Feature Sorting

*F*-score is a measure to distinguish the two types of samples [16], given that the training sample set *x*_*k*_ ∈ *R* ^*m*^,  *k* = 1,2,…, *n*, *l*  (*l* ≥ 2), is the number of the sample category and *n*_*j*_ is the sample number in the *j* class, *j* = 1,2,…, *l*. The *F*-score of *i* is defined in the training samples

(12) $$\begin{array}{c} F_{i}=\frac{\sum_{j=1}^{l}{\left({\overset{-}{x}}_{i}^{j}-{\overset{-}{x}}_{i}\right)}^{2}}{\sum_{j=1}^{l}\left(1/\left(n_{j}-1\right)\right)\sum_{k=1}^{n_{j}}{\left(x_{k,i}^{j}-{\overset{-}{x}}_{i}^{j}\right)}^{2}}, \end{array}$$

where ${\overset{-}{x}}_{i}$ is the average of the first *i* feature of the whole training set, ${\overset{-}{x}}_{i}^{j}$ is the average of the first *i* feature of the *j* class, and *x*_*k*,*i*_^*j*^ is the *i* feature of the first *k* sample data in *j* class.

### 3.2. Radar Chart for Feature Fusion

Radar graphic information is called graphical feature [17, 18]. Graphical features are the radar map feature area, focus feature, adjacent amplitude ratio, location characteristics, and zoning area ratio. The center of radar is an important visual characteristic, which can better respond to the internal relationship of each dimension characteristic.

An *M*-dimensional radar chart is constructed by sample data *r*_1_, *r*_2_,…, *r*_*i*_,…, *r*_*M*_, *M* polygon (*r*_*i*_, *r*_*i*+1_,…, *r*_*i*+*m*−1_) is composed of arbitrary continuous adjacent *m*-dimensional variables, and the center of *M* polygon by geometric algebra is

(13) absim=∑j=1mri+j−1cos⁡j−1∗w32+∑j=2mri+j−1sin⁡j−1∗w32,angleim=arctan⁡∑j=2mri+j−1sin⁡j−1∗w/32∑j=1mri+j−1cos⁡j−1∗w/32+2πi−1M,

where *w* = 2*π*/*M* is the angle of the adjacent features, abs_*im*_ is the amplitude of *M* polygonal center (*r*_*i*_, *r*_*i*+1_,…, *r*_*i*+*m*−1_), and  angle_*i*_ is angle direction of *M* polygonal center (*r*_*i*_, *r*_*i*+1_,…, *r*_*i*+*m*−1_).

### 3.3. Schema and Algorithm for Feature Optimization

**Figure 2:**
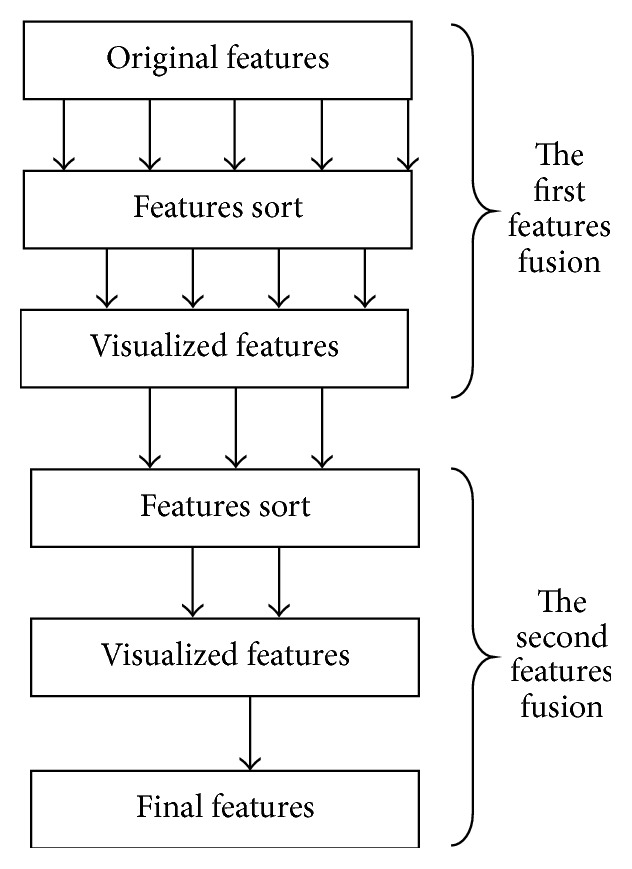
Features fusion and optimization.

In this work, we used the hierarchical visual technique for feature optimization. There are two hierarchical fusions in the process. In each level, firstly, the main aim is to sort the high-dimensional features. Secondly, the effecting features are got, which are grouped together as input to the next level, and the process is repeated to get fusion feature. Process is shown in Figure 2, where original features are (*x*_1_, *x*_2_,…, *x*_*i*_,…, *x*_*n*_), *x*_*i*_ is the first *i* feature, and *n* is feature dimension; the features fusion and reduction algorithm are as follows: input is original feature (*x*_1_, *x*_2_,…, *x*_*i*_,…, *x*_*n*_). Output is feature Re_fea after reduction.

(1) *F*-score value: *F*_*i*_ of *x*_*i*_ *F*-score according to formula (12).
(2) Feature sort: sort all features (*x*_1_, *x*_2_,…, *x*_*i*_,…, *x*_*n*_) by the *F*-score value; then (*x*_1_′, *x*_2_′,…, *x*_*i*_′,…, *x*_*n*_′) is got, and sort the *F*-score value (*F*_1_′, *F*_2_′,…, *F*_*i*_′,…, *F*_*n*_′), where *F*_1_′ ≥ *F*_2_′ ≥ ⋯≥*F*_*i*_′ ≥ ⋯≥*F*_*n*_′.
(3) Slicing: *F*_Mean is *F*-score average; the first *F*_first is less than *F*_Mean, so *F*_1_′ ≥ *F*_2_′ ≥ ⋯≥*F*_*F*_first−1_′ ≥ *F*_Mean, *F*_Mean < *F*_*F*_first_′; the first lay is (*x*_1_′, *x*_2_′,…, *x*_*F*_first−1_′); compute *F*_Mean2 of the average *F*-score from *F*_first to *n*; then *F*_second is less than *F*_mean2; the second level is (*x*_*F*_first_′, *x*_2_′,…, *x*_*F*_second−1_′); the third level is (*x*_*F*_second_′, *x*_*F*_second+1_′,…, *x*_*n*_′).
(4) Visual features fusion: specifically, in (13), if *m* = 2, to obtain the center of gravity when *m* = 2, *m* = 3, and *m* = 4, original feature set *S*′ is constructed by three feature set fusions.
(5) *S*′ is repeated to do steps (1), (2), and (3). Re_fea is got.

## 4. Pathologic Speech Intelligibility Evaluation

**Figure 3:**
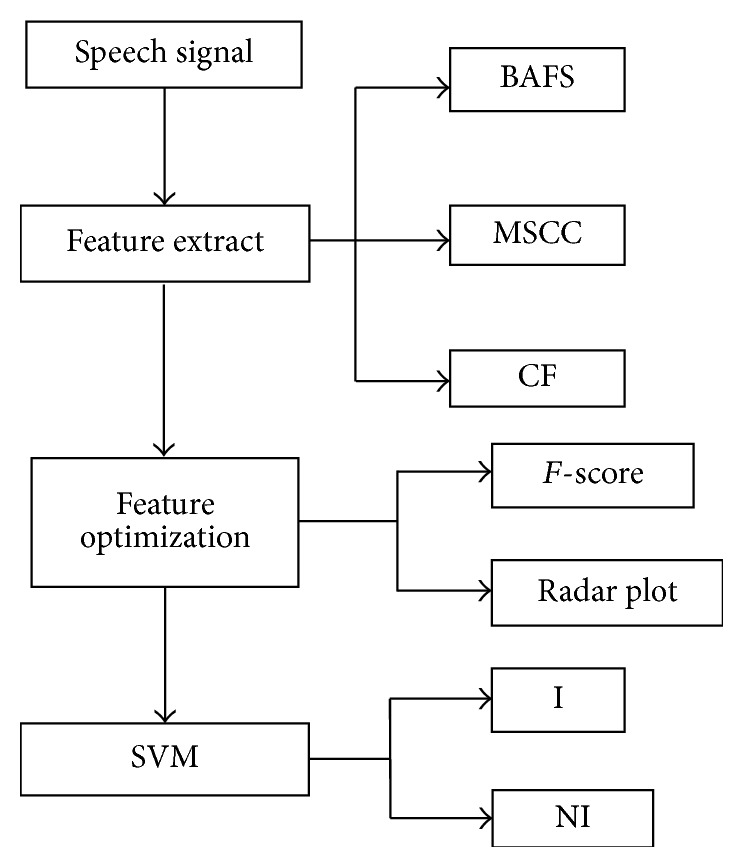
Speech intelligibility evaluation schema.

In Figure 3, firstly, pathological speech features are extracted from this system, including basic speech features, MSCC features, and nonlinear characteristics. Secondly, feature optimization is finished by means of *F*-score and radar chart. Finally, the speech intelligibility is evaluated by SVM classifier.

In the classification problems, SVM follows a certain procedure to find the separating hyperplane with the largest margin of two classes. Radial basis function (RBF), a kernel, is used in this article. The sensitivity, specificity, accuracy, and UA are an index. As a classification tool to evaluate the NKI-CCRT corpus by different feature sets, SVM algorithm constructs a set of reference vectors in role of boundaries that minimize the number of misclassifications. Therefore, it represents a low-cost, accurate, and automatic tool for pathological voice classification in contrast with other tools, such as Gaussian mixture model [19].

### 4.1. Corpus for Pathologic Speech Study

#### 4.1.1. NKI-CCRT Corpus

The NKI-CCRT corpus [14] is recorded by head and neck cancer surgery from the Netherlands Cancer Institute. 55 (10 males, 45 females) speakers are head and neck cancer patients undergoing chemotherapy, who are operated (CCRT) on in three stages (before treatment, after 10 weeks, and after 12 months). Recording mode is reading German neutral text. The 13 graduate or graduating language pathologists (average 23.7 years old) evaluated the intelligibility of their recordings. The evaluation index score is from 1 to 7. We get 13 statistics of each speaker's statement. INTERSPEECH 2012 speaker trait pathology challenge is divided into two categories according to statistics: I (intelligible) and NI (nonintelligible), where corpus sampling rate is 16 KH_Z_, quantified as 16 b. The corpus distribution is in Table 2.

#### 4.1.2. SVD Corpus

SVD [20] is the free pathological corpus in the Saarland University computation linguistics and phonetics laboratory. It is a collection of voice recordings from more than 2000 persons, where a session is defined as a collection of

1. recordings of vowels /a/, /i/, and /u/ produced at normal, high, low, and low-high-low pitch;
2. recordings of sentence “Guten Morgen, wie geht es Ihnen?” (“Good morning, how are you?”).

That makes a total of 13 files per session. In addition, the electroglottogram (EGG) signal is also stored for each case in a separate file. The length of the files with sustained vowels is between 1 and 3 seconds. All recordings are sampled at 50 kHz and their resolution is 16 bits. 71 different pathologies are contained, including both functional and organic. The corpus distribution is in Table 3.

### 4.2. Experimental Results

Further analysis is required to study the effect of various features of each subsystem.

#### 4.2.1. MSCC versus MFCC

*S*-transform is a time-frequency analysis method by Stock Well which combines the advantage of wavelet transform with short time Fourier transform [7], which shows better antinoise, time resolution, and time-frequency localization [21]. Therefore, in this paper, MSCC is proposed based on *S*-transform. MSCC is compared with the traditional MFCC in the NKI-CCRT and SVD corpus. Recognition results are shown in Tables 4 and 5. MSCC parameters improved significantly in the classification rate.

**Figure 4:**
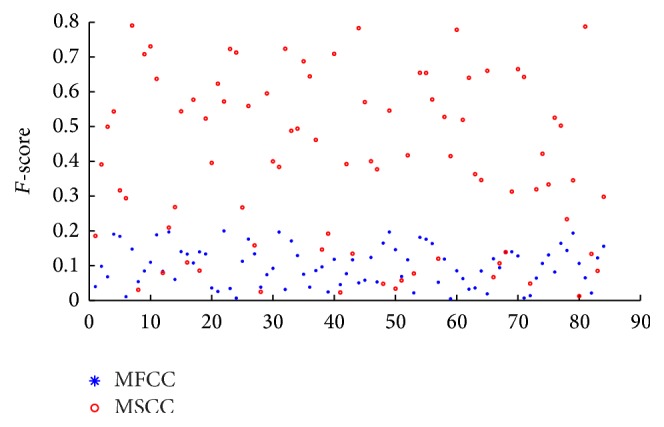
*F*-score of MSCC and MFCC.

For example, in NKI-CCRT corpus, each value index is improved, where UA is increased from 51.58% to 64.76% and accuracy is increased from 50.54% to 63.67%. Thus, MSCC contains more pathological information than MFCC. Meanwhile, in order to show the contrast that MSCC contains more information than MFCC directly, we use *F*-score values to evaluate MSCC and MFCC; MSCC shows better performance by *F*-score in Figure 4. The *x*-axis represents feature dimension. *y*-axis represents *F*-score values. MFCC is generally less than 0.2; the average is at 0.09. The maximum *F*-score of MSCC is nearly 0.8, and the average is about 0.39. The results of *F*-score indicate that the MSCC feature is stronger in the pathology classification.

#### 4.2.2. MSCC: Basis Acoustic Feature (BAFS) versus Chaotic Features (CF)

Firstly, MSCC is compared with basis acoustic features (430) by support vector machine (SVM). As it can be seen in Tables 6 and 7, the MSCC is better than BAFS in pathological speech intelligibility evaluation. Furthermore, it explains the effectiveness of the MSCC and BAFS feature set. Thirdly, the nonlinear characteristics of the pathological voice are considered as the supplement to pathological voice features. Chaotic features also have played a certain role and achieved a 58.16% recognition rate. But because the feature dimension is too small, the effect is not particularly obvious. The joint feature set (526) has the best performance.

#### 4.2.3. Feature Optimization

In our continued investigation, we design an automatic pathological speech intelligibility evaluation system by information visualization optimization method. Furthermore, this hierarchical method is experimented with in NKI-CCRT corpus. The classification accuracy of 84.44% can be achieved. Recognition results are shown in Table 8.

In our study, Table 8 shows that the fusion feature set Re_fea is more probable. It is obvious that Re_fea has sensitivity of 84.44% and 78.67%, which is higher than any other sensitivity. The result indicates that the Re_fea significantly improves voice disorder classification rate in comparison with other feature sets. Therefore, the hierarchical visual optimization method is effective and achieved better recognition rate than the baseline of INTERSPEECH 2012 challenge. The results from this experiment demonstrated that feature extraction method can be considered as a proper feature select strategy to increase identification accuracy of impaired voices.

## 5. Conclusion

The signal characteristics of pathological speech have been studied widely in the literature. A previous study showed that changes from articulatory manner are associated with pathological speech, while variability in articulatory place occurs to both normal and pathological speech. Therefore, the results of this research show that MSCC acoustic features fed to other pathology common features can be used together with invasive methods as complementary tools for pathological speech intelligibility evaluation. Furthermore, results of classification demonstrated that optimized feature set has great capability for classification of pathological voices to normal ones compared with the other feature that is examined in this research. Therefore, efficient combination of this work is composed of acoustic long-time features, MSCC, chaotic features, and SVM, which yield sensitivity of 84.4%. This structure significantly improves the results of pathological speech recognition in comparison with the proposed algorithm found in the references [22].

Feature extraction and pattern classification are a key of pathological speech recognition. This study proposes a new feature set and feature fusion method. The basis acoustic feature, precise time-frequency feature, and chaotic feature showed discriminating power for binary classification based fusion method (84.4% higher than the 79.9% of Kim et al. on the NKI-CCRT corpus [23]). Features fusion method shows significant improvement in classification accuracy from its original features set used. It shows that the pathological speech feature extraction and optimization were able to improve the performance of classification based on radar chart and *F*-score. Further analysis is required to study the effect of fusion difference classifiers. In addition, we would also like to study the effectiveness of other features and reduction methods like particle swarm optimization. In a word, the proposed method has greatly improved the pathological speech intelligibility evaluation performance and can provide important theoretical bases of the clinical application of speech pathology, which can be applied to other areas.

## Figures and Tables

**Table 1:** BAFS Feature Set Construction.

Types	Feature	Dimension
Prosodic features	fundamental frequency	15

Sound quality features	Jitter	15
	shimmer	15
	HNR	15

Related features based on spectral	Spectral Centroid	10
	Spectral Entropy	10
	Spectral Flux	10
	Spectral Asymmetry	10
	Spectral Slope	10
	Spectral Kurtosis	10
	Spectral Roll-off	40

**Table 2:** The NKI-CCRT corpus.

NCSC	Training set	Test set
I	384	341
NI	517	405

**Table 3:** The SVD corpus.

SVD	Training set	Test set
Healthy	434	198
Pathology	651	211

**Table 4:** MSCC and MFCC are compared based on NKI-CCRT corpus.

Feature	Sensitivity	Specificity	UA	Accuracy
MSCC	67.15%	62.36%	64.76%	63.67%
MFCC	56.25%	46.90%	51.58%	50.54%

**Table 5:** MSCC and MFCC are compared based on SVD corpus.

Feature	Sensitivity	Specificity	UA	Accuracy
MSCC	70.62%	69.20%	69.91%	68.95%
MFCC	61.61%	56.56%	59.09%	59.17%

**Table 6:** Basis acoustic feature and Chaotic features results based on NKI-CCRT corpus.

Feature	Sensitivity	Specificity	UA	Accuracy
BAFS (430)	63.70%	57.77%	60.74%	60.99%
CF (12)	55.31%	61.00%	58.16%	57.91%
MSCC + BAFS (514)	82.72%	65.10%	73.91%	74.66%
CF + MSCC + BAFS (526)	82.96%	65.68%	74.10%	75.07%

**Table 7:** Basis acoustic feature and Chaotic features results based on SVD corpus.

Feature	Sensitivity	Specificity	UA	Accuracy
BAFS (430)	73.93%	69.70%	69.91%	68.95%
CF (12)	62.56%	57.58%	60.07%	60.15%
MSCC + BAFS (514)	80.09%	71.21%	75.65%	75.79%
CF + MSCC + BAFS (526)	79.15%	73.23%	76.19%	76.28%

**Table 8:** Features optimization results.

Feature	Sensitivity	Specificity	UA	Accuracy
Re_fea (96- NKI-CCRT)	84.44%	65.69%	75.07%	75.87%
Re_fea (104- SVD)	78.67%	79.30%	78.99%	78.97%
Baseline (NKI-CCRT)	—	—	61.40%	—
